# Reply to ‘Pseudoreplication and greenhouse-gas emissions from rivers'

**DOI:** 10.1038/s41467-019-13304-0

**Published:** 2019-11-26

**Authors:** Sophie A. Comer-Warner, Paul Romeijn, Daren C. Gooddy, Sami Ullah, Nicholas Kettridge, Benjamin Marchant, David M. Hannah, Stefan Krause

**Affiliations:** 1 School of Geography, Earth and Environmental Sciences, University of Birmingham, Edgbaston, Birmingham, B15 2TT UK; 2 British Geological Survey, Maclean Building, Wallingford, Oxfordshire OX10 8BB UK

**Keywords:** Carbon cycle, Carbon cycle, Freshwater ecology

**Replying to** S. Tiegs et al. *Nature Communications* 10.1038/s41467-019-13303-1 (2019)

Tiegs et al.^1^ highlight the significance and relevance of the findings of Comer-Warner et al.^2^ on greenhouse-gas emissions from streambed sediments but raise questions about some aspects of the experimental design. We support their call for more detailed field and laboratory-based studies on this subject. However, we believe that their concerns relate to uncertainties and limitations in the experimental design that were discussed explicitly in the original paper (and accompanying transparent peer review process—available online), or represent criticisms related to highly improbable minor anomalies that may unnecessarily dismiss experimental results as discussed below.

It should be noted in a broader context that previous compelling articles have challenged arguments aligned to those of Tiegs et al.^1^ (and Hurlbert^3^, cited therein), which propagate the arbitrary dismissal of important research due to philosophical criticisms of pseudoreplication^4–7^. For instance, Davies et al.^4^ have shown that the exact formation of suitable hypotheses based on mechanistic understanding can account for pseudoreplication within experimental design. Without such underlying hypotheses the number of necessary potential controls are infinite and hence infeasible. Furthermore, problems of pseudoreplication may be reduced if appropriate statistics addressing the pseudoreplication are used, for example through inclusion as random effects in linear mixed effects models^7,8^. While we welcome the contribution of Tiegs et al.^1^ to this longstanding discourse, our response aims particularly at those elements that advance the discussion beyond a repetition of previous pseudoreplication controversies^1,3–7^.

The research design of Comer-Warner et al.^2^ was based on a hypothetico-deductive approach that focused on the key predictors (i.e. controls and proxies for processes) derived from state-of-the-art understanding of our target variables (CO_2_ and CH_4_ emissions). This research design provided a framework for robust statistical analysis through clearly defined hypothesised processes and mechanisms in order to support meaningful statistical analyses.

Tiegs et al.^1^ express concerns that Comer-Warner et al.^2^ included all samples within the same batch in the same incubator. We did not account for week of incubation (i.e. batch) within the statistical model as we do not consider that there is a reasonable mechanism by which sample incubation week was impacted by this experimental approach and the associated consistent sample storage over the course of batch incubations. We find Tiegs et al.^1^ argumentation that batch-specific conditions “likely differed in unknown ways” from batches tested in other weeks highly unconvincing. Moreover, we do not consider isolative segregation to have any discernible impact on the experimental results, as discussed below. In fact, we are convinced that the results of our experiments are more robust by exposing all experimental temperature treatments to the same incubation environment, rather than introducing unnecessary uncertainty and risk of technical failure or variance in performance through the use of different incubators, as suggested by Tiegs et al.^1^. Notably, the differences between the temperature at the top and bottom of the incubator were very small (0.0 to 0.6 °C, Table 1), within the typical error range of standard electronic temperature measurement devices, further indicating that the incubator provided uniform environmental conditions.

**Table 1:** The variation in temperature between the top and bottom of the incubator used.

Temperature treatment (°C)	Mean difference (°C)	Standard deviation (°C)
5	0.6	0.3
9	0.6	0.2
15	0.4	0.2
21	0.1	0.1
26	0.0	0.2

Tiegs et al.^1^ furthermore highlight the lack of replication of geology as a treatment and posit that no conclusions can be drawn with respect to geological effects in Comer-Warner et al.^2^. We would like to emphasise that our conclusions at no point claim to draw interpretations for the entirety of the two example geologies used in Comer-Warner et al.^2^. Instead, we followed a paired catchment approach as has been used for more than 100 years in hydrological and environmental sciences^9,10^, using the observed differences between multiple samples from different locations in each stream to highlight the differences between the two rivers, which varied predominantly by geology.

While we agree with Tiegs et al.^1^ about the potential uncertainties arising from the storage of sediments at 4 °C during the course of the experiments as discussed in detail in Comer-Warner et al.^2^, we consider the alternative of repeated sampling closer to the time of respective batch incubations to impose considerably larger experimental uncertainties due to the temporally highly dynamic nature of river and streambed chemical and microbial conditions, with biogeochemical turnover ranging from minutes to days. Based on our sampling strategy and similar starting points for the treatment effects, our statistical analysis showed significant differences between the two streams over the course of independent batch incubations.

Additionally, Tiegs et al.^1^ state that the conclusion of non-linear and threshold responses observed in our data are predominantly based on the reduction in microbial activity observed from 21 to 26 °C. We would like to highlight that this statement is not accurate as a decrease in microbial activity was not observed between 21 and 26 °C in all sediment classes and was not observed in the case of CO_2_ production in any sediment classes. The interpretation of non-linear and threshold responses was, therefore, not solely reliant on the observation of lower microbial activity at 26 than 21 °C, as suggested by Tiegs et al.^1^. Tiegs et al.^1^ express scepticism about the threshold responses observed in Comer-Warner et al.^2^ and cite the supposed linear relationship between methane fluxes and temperature determined from the meta-analysis of Yvon-Durocher et al.^12^. Tiegs et al.’s^1^ account of the results presented in Yvon-Durocher et al.^11^ is not accurate though, as their meta-analysis found exponential and non-linear relationships between methane fluxes and temperature (as highlighted in the Addendum for Comer-Warner et al.^2^). Furthermore, non-linearity and threshold responses of greenhouse-gas fluxes to temperature have previously been found in a variety of ecosystems, e.g., refs. ^12-14^.

## Data Availability

The dataset generated during the current study is available from the corresponding author on reasonable request.
